# A Bill for the Doctor’s Benefit

**Published:** 1898-02

**Authors:** 


### A Bill for the Doctor's Benefit.

An esteemed subscriber in renewing his subscription writes:

*Editor Texas Medical Journal:*

I hope you will continue the fight on quackery and for an honorable medical profession in Texas. It seems to me that if the profession would unite we might bring sufficient pressure to bear on the legislature to induce them to enact a law to exclude incompetent persons and repeal the robber occupation tax law. I think, however, that the legislation we need most is such as would enable a doctor to collect a reasonable fee, occasionally, for his services.

The leading surgeons and specialists who, by the way, usually have the most influence in directing the action of our Associations, perhaps do not so much feel the need of this kind of protection. Their clientelle is such and the nature of their work is such, that probably they are well paid. But it is not so with the general practitioner, and especially in the country. The patient may be ever so willing to pay, but the landlord's lien and the merchant's mortgage stand in the way. Shylock demands his pound of flesh, and though the doctor's services were as much a necessity as anything furnished by landlord or merchant, he gets nothing.

What we need most is a law that will exempt a small amount from these liens and set it aside for medical services. Such a law would work no hardship or injustice to any man; a law that exempted ten dollars for each family would have been worth $1000 to me this fall, and those that paid it would have been just as well off as they are, for somebody got all they had, anyway.

What I want is a fair division; I have just as much right to what is mine as the landlord to his rent, and if the law will give it to me I am willing to pay $75 occupation tax. Why not have a landlord's occupation tax? Let us see what others think of this suggestion. Yours fraternally, A. J. F.

